# Research on the Association Between Obstructive Sleep Apnea Hypopnea Syndrome Complicated With Coronary Heart Disease and Inflammatory Factors, Glycolipid Metabolism, Obesity, and Insulin Resistance

**DOI:** 10.3389/fendo.2022.854142

**Published:** 2022-07-01

**Authors:** Yumei Wen, Haibin Zhang, Yu Tang, Rui Yan

**Affiliations:** Department of Cardiology, Beijing Luhe Hospital, Capital of Medical University, Beijing, China

**Keywords:** obstructive sleep apnea hypopnea syndrome, coronary heart disease, obesity, inflammation, glycolipid metabolism

## Abstract

The aim of this study is to explore the association between obstructive sleep apnea hypopnea syndrome (OSAHS) complicated with coronary heart disease (CHD) and inflammatory factors, glycolipid metabolism, obesity, and insulin resistance. A total of 400 patients diagnosed with OSAHS who underwent polysomnography (PSG) monitoring in the Sleep Diagnosis and Treatment Center of Beijing Luhe Hospital from March 2015 to September 2018 were selected and divided into the OSAHS group (*n* = 200) and the OSAHS + CHD group (*n* = 200) according to disease condition. The questionnaire survey was conducted, the somatology indexes were measured, and the PSG, insulin, glycolipid metabolism parameters, and serum inflammatory factors were detected. Body weight, body mass index, waist circumference, and Epworth sleepiness scale (ESS) score were all significantly increased in the OSAHS + CHD group compared with those in the OSAHS group (*p* < 0.05). The microarousal index (MAI), apnea hypopnea index (AHI), cumulative percentage of time spent at oxygen saturation below 90% (CT90%), oxygen desaturation index (ODI), lowest oxygen saturation (LSaO_2_), total apnea time (TAT), and mean oxygen saturation (MSaO_2_) had statistically significant differences between the OSAHS + CHD group and the OSAHS group (*p* < 0.05). According to the Spearman correlation analysis of AHI, LSaO_2_, MSaO_2_, CT90%, ODI, and MAI with HOMA-IR in both groups, MAI, AHI, CT90%, and ODI were positively correlated with HOMA-IR (*r* > 0), while LSaO_2_ and MSaO_2_ were negatively correlated with HOMA-IR (*r* < 0). Compared with the OSAHS group, the OSAHS + CHD group had an obviously increased level of triglyceride (TG) (*p* < 0.05), and obviously increased levels of serum inflammatory factors C-reactive protein (CRP), tumor necrosis factor-α (TNF-α), interleukin-6 (IL-6), and interferon-γ (IFN-γ) (*p* < 0.05). The occurrence of OSAHS complicated with CHD is related to inflammatory factors, glycolipid metabolism, obesity rate, and HOMA-IR.

## Introduction

Obstructive sleep apnea hypopnea syndrome (OSAHS), whose morbidity rate is annually increasing, is mainly manifested as sleep structure disorder, drowsiness, breath blockage, apnea, snoring, etc. (1), which increases the risks of cardiovascular complications and multiple organ damage in patients. OSAHS not only harms the respiratory system but also affects the cardio-cerebrovascular system of patients, and causes such complications as heart failure and coronary heart disease (CHD), seriously affecting the quality of life and lifespan of patients (2, 3). OSAHS has a high morbidity rate in middle-aged and elderly people, and it is also affected by genetics, unhealthy living habits, and social pressure (4). CHD is caused by the myocardial tissue lesions due to stenosis or obstruction of the arterial lumen (5). CHD has become a common cardio-cerebrovascular disease in recent years, and it frequently occurs in the elderly, but patients have become increasingly younger nowadays, with an increasing annual morbidity rate (6). The etiology of CHD is complex, and the effective prevention and control of its risk factors has an important significance to reduce its morbidity rate (7). CHD seriously threatens human health; thus, it has gradually attracted widespread attention in society (8). According to a large number of studies in China and other countries, OSAHS is closely related to CHD (9). Studies have also shown that the morbidity rate of OSAHS in CHD patients is higher than that in the general population, and OSAHS complicated with CHD will raise the mortality rate (10). Therefore, this paper conducted a questionnaire survey of patients with CHD, measured somatic indexes, and detected polysomnography (PSG), insulin, glucose and lipid metabolism parameters, and serum inflammatory factors, so as to analyze the expression differences in inflammatory factors, and glucose and lipid metabolism in patients with OSAHS complicated with CHD. Insulin resistance (HOMA-IR) was evaluated by obesity rate and a homeostasis model to explore the relationship between OSAHS combined with CHD and inflammatory factors, glucose and lipid metabolism, obesity, and IR of cardiovascular disease.

## Objects and Methods

### Objects of the Study

A total of 400 patients diagnosed with OSAHS who underwent PSG monitoring in the Sleep Diagnosis and Treatment Center of Beijing Luhe Hospital from March 2015 to September 2018 were selected and divided into the OSAHS group (*n* = 114) and the OSAHS + CHD group (*n* = 286) according to disease condition.

### Inclusion and Exclusion Criteria

Inclusion criteria were as follows: patients with such clinical symptoms as loud snoring with apnea during nighttime sleep, mouth breathing, frequent urination at night, mouth dryness in the morning, memory deterioration, and daytime sleepiness [Epworth sleepiness scale (ESS) score ≥9 points]; those with an apnea hypopnea index (AHI) ≥ 5 times/h according to PSG; those with upper airway stenosis and obstruction shown in the examination; those diagnosed with OSAHS; and those with an ESS score <9 points but AHI ≥5 times/h. According to the international common visual diameter method, the degree of vascular stenosis = (normal vessel diameter near the heart of the stenosis site − diameter of the stenosis site)/vessel diameter near the heart of the stenosis segment × 100%, and coronary angiography showed that the stenosis degree of the inner diameter of the main branches was more than 50%, which could also be diagnosed as OSAHS complicated with CHD. Exclusion criteria were as follows: patients with upper airway resistance syndrome, simple snoring, or narcolepsy; those with a clear history of diabetes earlier than OSAHS; those with IR caused by other factors, such as pancreatic exocrine disease or endocrine diseases; and those with severe malformations of the mouth, nose, or neck.

### Questionnaire Survey and Measurement of Somatology Indexes

The main contents of questionnaire survey are as follows:

1. Basic information: gender and age.
2. Past medical history: with or without a history of upper respiratory diseases (such as acute and chronic rhinitis, sinusitis, pharyngitis, angina, severe sinusitis, and deviation of nasal septum), cardiovascular/cerebrovascular diseases, hypertension, and diabetes.
3. Personal history: with or without a history of smoking and alcoholism.
4. Family history: with or without a family history of OSAHS, heart disease, and diabetes.
5. ESS score: The daytime sleepiness was scored in all subjects according to the ESS (**Table 1**).

**Table 1:** ESS.

Are you sleepy in the following conditions?	Never	Rarely	Sometimes	Often
Sit reading books				
Watch TV				
Sit still somewhere				
Long-time riding (>1 h)				
Lie down for rest at noon				
Sit chatting with people				
Sit quietly at noon without drinking				
Stop at the traffic light in traffic jam				

Never, 0 point; rarely, 1 point; sometimes, 2 points; often, 3 points. "Never": not occur at all, "Rarely": once-twice a week, "Sometimes": 3-4 times a week, "Often": more than 5 times a week.

Somatology indexes are as follows:

1. Body weight: After fasting, patients wore thin clothes, took off their shoes, and urinated before their body weight (kg) was measured using a weighing scale and recorded.
2. Waist circumference: the circumference (cm) of the plane at the midpoint between the inferior margin of rib and the crista iliaca.
3. Height: The patients kept their body upright and their heels were placed together, and then the height (cm) was measured and recorded.
4. Body mass index (BMI) = weight/height^2^ (kg/m^2^).
5. Waist-to-height ratio (WHtR) = waist circumference (cm)/height (cm).

### PSG Monitoring

In the daytime, the patients did not sleep without eating food containing stimulants and sedatives that affected sleep. The sleep process (more than 7 h overnight) was monitored *via* PSG from 9:00 p.m. to 8:00 a.m. the next day. The following indexes were recorded and analyzed: cumulative percentage of time spent at oxygen saturation below 90% (CT90%), oxygen desaturation index (ODI), microarousal index (MAI), AHI, lowest oxygen saturation (LSaO_2_), mean oxygen saturation (MSaO_2_), longest apnea time (LAT), and total apnea time (TAT).

### Detection of Fasting Plasma Glucose and Fasting Insulin

After fasting for more than 8 h overnight, the peripheral venous blood (elbow venous blood samples) was drawn in the morning to detect FPG and FINS through hexokinase assay and chemiluminescence assay. The IR level was evaluated using homeostasis model assessment of IR (HOMA-IR): HOMA-IR = FINS (mIU/ml) × FPG (mmol/L)/22.5.

### Detection of Glycolipid Metabolism Parameters

After fasting for more than 8 h overnight, the peripheral venous blood (elbow venous blood samples) was drawn in the morning to routinely detect FPG, total cholesterol (TC), triglyceride (TG), high-density lipoprotein (HDL), and low-density lipoprotein (LDL).

### Detection of Serum Inflammatory Factors

After fasting overnight, 3–4 ml of venous blood was drawn from patients and centrifuged at 3,000 rpm for 15 min. Then, the supernatant was taken using a micropipettor and stored at −80°C. The levels of C-reactive protein (CRP), tumor necrosis factor-α (TNF-α), interleukin-6 (IL-6), and interferon-γ (IFN-γ) in both groups were determined repeatedly 3 times *via* enzyme-linked immunosorbent assay (ELISA) according to the instructions, and the results were averaged.

### Statistical Methods

SPSS 20.0 software was used for statistical analysis. The differences in inflammatory factors, glycolipid metabolism, obesity rate, and HOMA-IR were compared between the two groups using *t*-test. *p* < 0.05 suggested that the difference was statistically significant.

## Results

### Clinical Data

Body weight, BMI, waist circumference, and ESS score were all found to be significantly increased in the OSAHS + CHD group compared with those in the OSAHS group, and the differences were statistically significant (*p* < 0.05), while other indexes had no statistically significant differences between the two groups (*p* > 0.05) (**Table 2**).

**Table 2:** Clinical data in OSAHS + CHD group and OSAHS group.

Item	OSAHS group	OSAHS + CHD group	χ2	*p*
Age (years old)	45.68±7.56	47.25±8.71	0.03	0.830
Weight (kg)	74.12±11.95	82.78±10.69	6.47	0.006
BMI (kg/m2)	25.48±1.98	29.34±2.14	4.02	0.005
Waist circumference (cm)	98.15±12.35	102.49±13.78	3.08	0.003
WHtR	0.55±0.01	0.56±0.02	0.06	0.745
ESS score	8.56±3.24	11.23±4.19	5.47	0.029
Gender (male/female)	112/88	146/54	4.48	0.092
Smoking history	95	103	3.32	0.126
Drinking history	82	98	1.42	0.623

### PSG Monitoring Results in Both Groups

MAI, AHI, CT90%, ODI, LSaO_2_, TAT, and MSaO_2_ had statistically significant differences between the OSAHS + CHD group and the OSAHS group (*p* < 0.05) (**Table 3**).

**Table 3:** PSG monitoring results in both groups.

Item	OSAHS group	OSAHS + CHD group	χ2	*p*
AHI (times/h)	16.48±3.46	51.86±9.48	24.69	0.003
LSaO2 (%)	88.46±10.65	61.87±13.49	13.45	0.004
CT90% (%)	5.14±7.26	38.16±4.23	14.26	0.005
ODI (times/h)	19.16±4.03	35.09±9.61	10.03	0.005
MSaO2 (%)	96.05±2.04	88.74±1.95	7.12	0.004
TAT (min)	40.38±190.23	164.29±20.46	19.45	0.002
MAI (times/h)	20.31±9.01	34.16±12.73	6.09	0.004

### HOMA-IR

**Figure 1:**
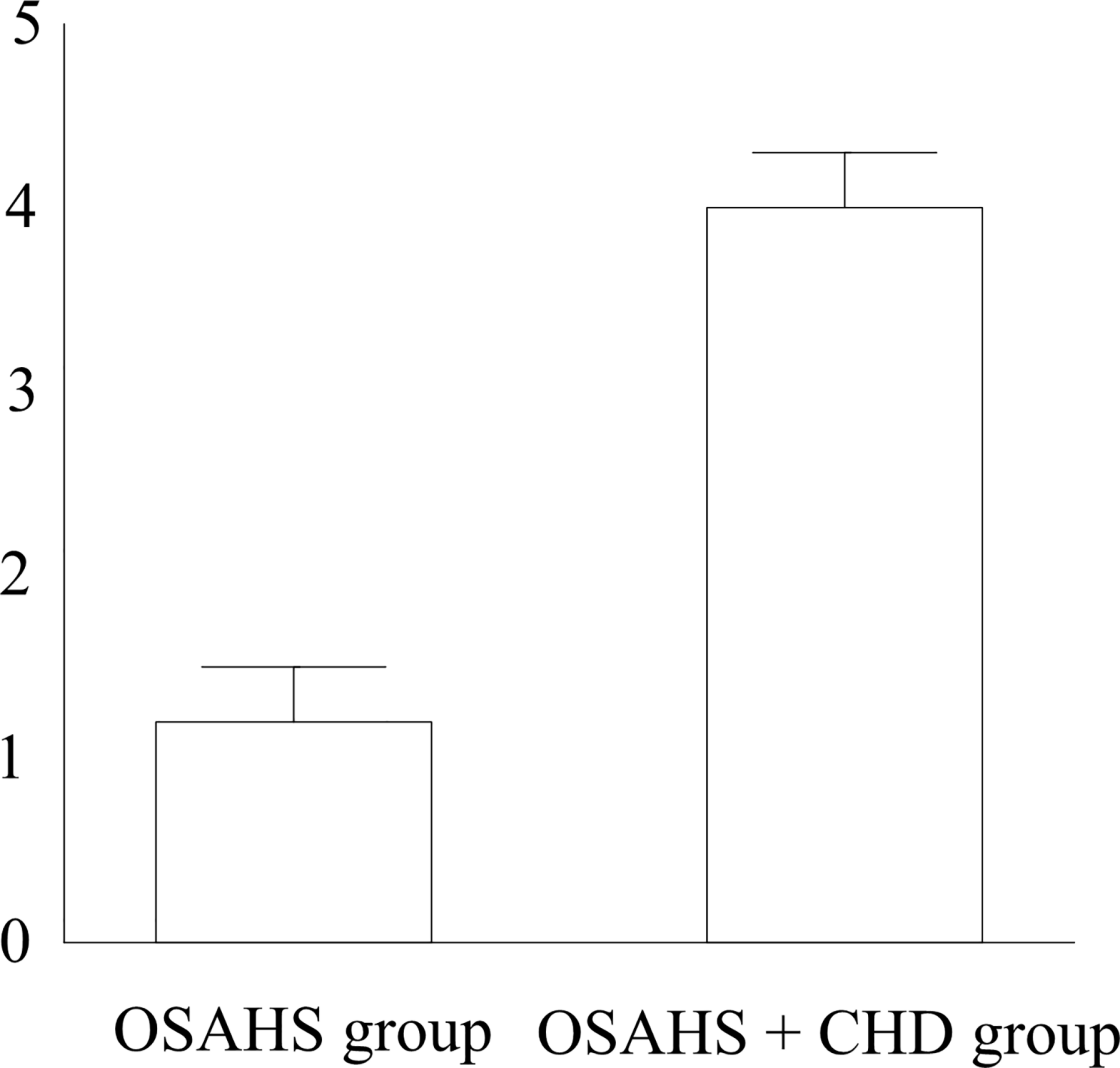
HOMA-IR. & *p* < 0.05 vs. the OSAHS group.

HOMA-IR was the highest (4.65 ± 0.71) in the OSAHS + CHD group and the lowest (1.24 ± 0.25) in the OSAHS group (*p* < 0.05) (**Figure 1**). According to the Spearman correlation analysis of AHI, LSaO_2_, MSaO_2_, CT90%, ODI, MAI, and TAT with HOMA-IR in both groups, AHI, CT90%, ODI, MAI, and TAT were positively correlated with HOMA-IR (*r* > 0), while LSaO_2_ and MSaO_2_ were negatively correlated with HOMA-IR (*r* < 0) (**Table 4**).

**Table 4:** Correlation analysis of variables with HOMA-IR.

Item	Weight	BMI	Waist circumference	ESS score	AHI	LSaO2	MSaO2	CT90%	ODI	MAI	TAT
*r*	0.16	0.15	0.18	0.21	0.83	-0.25	-0.22	0.82	0.12	0.18	0.35
*p*	0.002	0.002	0.001	0.002	0.001	0.001	0.002	0.004	0.013	0.001	0.13

### Glycolipid Metabolism Parameters

There were no obvious differences in the FPG, TC, HDL, and LDL between the OSAHS + CHD group and the OSAHS group (*p* > 0.05). Compared with the OSAHS group, the OSAHS + CHD group had an obviously increased level of TG, showing a statistically significant difference (*p* < 0.05) (**Table 5**).

**Table 5:** Glycolipid metabolism parameters.

Item	OSAHS group	OSAHS + CHD group	*p*
FPG (mmol/L)	4.92±0.08	5.95±0.12	0.061
TC (mmol/L)	4.19±0.35	5.81±0.25	0.076
TG (mmol/L)	1.43±0.07	6.61±0.43	0.016
HDL (mmol/L)	1.59±0.15	1.06±0.36	0.091
LDL (mmol/L)	3.42±0.04	2.98±0.13	0.106

### Serum Inflammatory Factors

Compared with the OSAHS group, the OSAHS + CHD group had obviously increased levels of serum inflammatory factors CRP, TNF-α, IL-6, and IFN-γ, and there were also statistically significant differences (*p* < 0.05) (**Table 6**).

**Table 6:** Content of serum inflammatory factors in both groups.

Item	OSAHS group	OSAHS + CHD group	*p*
CRP	2.38±1.08	15.30±2.14	0.009
TNF-α	21.91±4.25	39.16±3.94	0.010
IL-6	8.19±3.49	13.07±1.80	0.005
IFN-γ	76.09±15.55	104.06±20.72	0.003

## Discussion

There are at least over 50 million patients with OSAHS in China, and most of them receive no active and effective treatment. On the one hand, patients regard “snoring” as a normal phenomenon rather than a disease. On the other hand, there is still a lack of effective biological indexes for early detection of OSAHS-related complications currently (11–14). Therefore, raising the patient’s awareness of the disease and its complications, and strengthening the early detection of OSAHS-related complications contribute to the treatment of disease and improvement of the prognosis of patients (15). The pathogenesis of OSAHS is upper airway obstruction (16), and OSAHS patients have neurohumoral abnormality (17). OSAHS can cause lesions in the cardiovascular system, and different types of CHD and OSAHS can also promote each other; thus, there is a complicated pathophysiological relation between OSAHS and CHD (18).

In the present study, body weight, BMI, waist circumference, and ESS score were all significantly increased in the OSAHS + CHD group compared with those in the OSAHS group, and the differences were statistically significant (*p* < 0.05). MAI, TAT, AHI, CT90%, ODI, LSaO_2_, and MSaO_2_ had obvious differences between the OSAHS + CHD group and the OSAHS group (*p* < 0.05). There were no obvious differences in the FPG, TC, HDL, and LDL between the OSAHS + CHD group and the OSAHS group (*p* > 0.05). Compared with the OSAHS group, the OSAHS + CHD group had an obviously increased level of TG, and there was a statistically significant difference (*p* < 0.05). To sum up, OSAHS has a significant correlation with obesity. OSAHS patients have such symptoms as daytime sleepiness, greasy food preference, and fat accumulation, which will aggravate the obesity of patients. The number of obesity patients with OSAHS + CHD is remarkably larger than those with OSAHS. Therefore, increasing the amount of exercise and reducing the intake of excessive high-calorie food can effectively improve the symptoms of OSAHS. In this study, among the glycolipid metabolism parameters, only TG had obvious changes in OSAHS + CHD compared with OSAHS, but no remarkable changes were observed in other parameters. Such a result is consistent with the conclusion made by Qing who used the SAS score and 4 different kinds of questionnaires as the screening tools for OSAHS such that the excessive fat accumulation in the thorax due to obesity leads to a decline in chest wall compliance and an increase in respiratory muscle resistance, which are most apparent in a supine position, and hypoxemia occurs in severe cases, forming a vicious cycle (19).

In this study, HOMA-IR was the highest in the OSAHS + CHD group and the lowest in the OSAHS group (*p* < 0.05). According to the Spearman correlation analysis of AHI, LSaO_2_, MSaO_2_, CT90%, ODI, and MAI with HOMA-IR in both groups, AHI, CT90%, ODI, and MAI were positively correlated with HOMA-IR (*r* > 0), while LSaO_2_ and MSaO_2_ were negatively correlated with HOMA-IR (*r* < 0). Moreover, compared with the OSAHS group, the OSAHS + CHD group had obviously increased levels of serum inflammatory factors CRP, TNF-α, IL-6, and IFN-γ, displaying statistically significant differences (*p* < 0.05). Hotamisligil et al. studied and found that IR is closely related to plasma pro-inflammatory factors, and the enhanced IR corresponds to the increased secretion of pro-inflammatory cytokines. Due to the increase in respiratory resistance, the patient experiences hypoxia, and a large number of inflammatory cytokines are expressed, thereby increasing the concentration of inflammatory factors in plasma. Such a result is consistent with the conclusion made by Cai et al. in their study on respiratory resistance in OSAHS patients that there is an inseparable relationship between OSAHS and IR, heart disease, and inflammatory factors, which demonstrates that detection of IR, heart disease, and inflammatory factors in patients may have an important significance for the prevention and treatment of OSAHS (20).

In conclusion, glycolipid metabolism, obesity rate, HOMA-IR, and inflammatory factors are all significantly increased in OSAHS + CHD compared with OSAHS.

## Data Availability Statement

The original contributions presented in the study are included in the article/supplementary material. Further inquiries can be directed to the corresponding author.

## Ethics Statement

The study was approved by the ethics committee of Beijing Luhe Hospital, and written informed consents were signed by the patients and/or guardians. The patients/participants provided their written informed consent to participate in this study.

## Author Contributions

YW wrote the manuscript. YW and HZ were responsible for questionnaire survey and measurement of somatology indexes. YT and RY performed ELISA. All authors contributed to the article and approved the submitted version.

## Conflict of Interest

The authors declare that the research was conducted in the absence of any commercial or financial relationships that could be construed as a potential conflict of interest.

## Publisher’s Note

All claims expressed in this article are solely those of the authors and do not necessarily represent those of their affiliated organizations, or those of the publisher, the editors and the reviewers. Any product that may be evaluated in this article, or claim that may be made by its manufacturer, is not guaranteed or endorsed by the publisher.
